# Digital image analysis of multiplex fluorescence IHC in colorectal cancer recognizes the prognostic value of CDX2 and its negative correlation with SOX2

**DOI:** 10.1038/s41374-019-0336-4

**Published:** 2019-10-22

**Authors:** Nair Lopes, Christian Holst Bergsland, Merete Bjørnslett, Teijo Pellinen, Aud Svindland, Arild Nesbakken, Raquel Almeida, Ragnhild A. Lothe, Leonor David, Jarle Bruun

**Affiliations:** 1 Department of Molecular Oncology, Institute for Cancer Research, Oslo University Hospital, Oslo, Norway; 2 i3S—Institute for Research and Innovation in Health, University of Porto, Porto, Portugal; 3 IPATIMUP—Institute of Molecular Pathology and Immunology, University of Porto, Porto, Portugal; 4 K.G. Jebsen Colorectal Cancer Research Centre, Division of Cancer Medicine, Oslo University Hospital, Oslo, Norway; 5 Institute for Clinical Medicine, Faculty of Medicine, University of Oslo, Oslo, Norway; 6 Institute for Molecular Medicine Finland (FIMM), University of Helsinki, Helsinki, Finland; 7 Department of Gastrointestinal Surgery, Oslo University Hospital, Oslo, Norway; 8 Faculty of Medicine, University of Porto, Porto, Portugal; 9 Department of Biology, Faculty of Sciences, University of Porto, Porto, Portugal

**Keywords:** Prognostic markers, Colorectal cancer, Fluorescence imaging, Immunohistochemistry, Translational research

## Abstract

Flourescence-based multiplex immunohistochemistry (mIHC) combined with multispectral imaging and digital image analysis (DIA) is a quantitative high-resolution method for determination of protein expression in tissue. We applied this method for five biomarkers (CDX2, SOX2, SOX9, E-cadherin, and β-catenin) using tissue microarrays of a Norwegian unselected series of primary colorectal cancer. The data were compared with previously obtained chromogenic IHC data of the same tissue cores, visually assessed by the Allred method. We found comparable results between the methods, although confirmed that DIA offered improved resolution to differentiate cases with high and low protein expression. However, we experienced inherent challenges with digital image analysis of membrane staining, which was better assessed visually. DIA and mIHC enabled quantitative analysis of biomarker coexpression on the same tissue section at the single-cell level, revealing a strong negative correlation between the differentiation markers CDX2 and SOX2. Both methods confirmed known prognostic associations for CDX2, but DIA improved data visualization and detection of clinicopathological and biological associations. In summary, mIHC combined with DIA is an efficient and reliable method to evaluate protein expression in tissue, here shown to recapitulate and improve detection of known clinicopathological and survival associations for the emerging biomarker CDX2, and is therefore a candidate approach to standardize CDX2 detection in pathology laboratories.

## Abstract (web summary)

Digital image analysis (DIA) of multiplex fluorescence-based immunohistochemistry and visual chromogenic evaluation of CDX2, SOX2, SOX9, E-cadherin, and β-catenin in colorectal cancer are comparable, recognizing prognostic value of CDX2 and negative correlation with SOX2. Membrane staining is best evaluated visually, while DIA enables single-cell coexpression analysis and improves visualization and detection of clinicopathological and biological associations.

## Introduction

Protein expression of biomarkers in cancer tissue is routinely assessed by immunohistochemistry (IHC) and relies on visual and semiquantitative evaluation of staining patterns and intensity. IHC is easy to perform and does not require advanced or expensive equipment, making it accessible to almost every laboratory. The study of biomarkers in large patient series was greatly facilitated by the development of the tissue microarray (TMA) technology [1, 2]. However, for the majority of biomarkers there are no standard criteria used for the manual scoring and subsequent semiquantitative analysis of protein expression, making characterization at different subcellular locations a subjective and potentially complex task [3]. The Allred score [4], which summarizes the intensity and the extent of staining, and the H-score which multiplies them [5] are two widely used methods. Consequently, patients are divided into different subgroups depending on which scoring system is used and the results are therefore not directly comparable. Furthermore, technical variations in antibody concentration and detection systems have a major impact on the intensity of staining, particularly when using chromogenic IHC, which has a very limited linear dynamic signal range, with significant consequences for downstream analyses [6]. Moreover, for high-throughput studies and when single-cell analysis is relevant, visual scoring of chromogenic IHC is time consuming and, in most cases, not feasible [7].

An alternative method that can address many of these problems is fluorescence-based multiplex IHC (mIHC) combined with multispectral imaging and digital image analysis (DIA). mIHC is based on the interrogation of multiple antigens on the same tissue section and multispectral imaging enables unmixing of several different fluorescent spectra, including tissue autofluorescence [8, 9]. The signal obtained by fluorescent detection has a much larger linear dynamic range than chromogenic detection, providing the basis for a more precise and objective quantification of protein expression [6, 10, 11].

In this study, we aimed to identify benefits and drawbacks with DIA and mIHC in comparison with conventional IHC analyzed according to the Allred method for colorectal cancer research and clinical use, with a particular focus on the clinically relevant markers CDX2 and SOX2. We performed DIA of fluorescence-based mIHC stains of CDX2, SOX2, SOX9, E-cadherin, and β-catenin in colorectal cancer samples arranged in TMAs, and compared with previous visually scored chromogenic IHC stains of the same series [12, 13]. All five markers are related to tumor differentiation and tumor stemness, and expected to be largely expressed in both normal mucosa (except for SOX2) and in epithelial cancer cells of the colorectum, and only infrequently in stromal cells; CDX2, SOX2, and SOX9 staining were expected to localize to the nucleus and to some degree also to the cytoplasm, E-cadherin was expected to localize to the membrane and the cytoplasm, and β-catenin should frequently be expressed in all three cellular compartments (proteinatlas.org). Finally, we compared clinicopathological associations obtained by the two methods focusing on the prognostic assessment of CDX2 and its inverse correlation with SOX2.

## Materials and methods

### Patient samples

During a 10-year period (1993–2003) 1290 patients were diagnosed with primary colorectal cancer at Oslo University Hospital—Aker hospital site (Norway), of whom 927 underwent major resection and tumor samples were included on a TMA (one 0.6 mm tissue core from central tumor per patient) distributed on four receiver blocks, as described previously [12]. Aker hospital served a geographically defined catchment area with a population of about 270,000 inhabitants in this period and the cohort is population representative for the Oslo area. Relevant clinical data was collected prospectively, analyzed retrospectively, and recorded in a local database which was quality controlled at follow-ups. Our data was cross-checked with the Cancer Registry of Norway which records data on all patients diagnosed with CRC. Microsatellite instability (MSI) status was determined using the consensus markers suggested by the National Cancer Institute, as described previously [14] (Table 1).

**Table 1:** Patient characteristics for cases included in prognostic comparison for CDX2 protein expression with Allred and DIA scoring

	Allred			DIA		
	CDX2+ (*n* = 526)	CDX2− (*n* = 63)	*P* value	CDX2+ (*n* = 522)	CDX2− (*n* = 67)	*P* value
Gender			0.32			0.94
Female	264 (50)	33 (52)		255 (49)	42 (63)	
Male	262 (50)	30 (48)		267 (51)	25 (37)	
Age			0.59			0.81
30–50	30 (6)	4 (6)		29 (6)	5 (7)	
51–70	180 (34)	22 (35)		179 (34)	23 (34)	
71–96	316 (60)	37 (59)		314 (60)	39 (58)	
Tumor stage			0.33			0.35
1	82 (16)	5 (8)		84 (16)	3 (4)	
2	220 (42)	26 (41)		216 (42)	30 (45)	
3	136 (26)	16 (25)		133 (26)	19 (28)	
4	85 (16)	16 (25)		86 (17)	15 (22)	
pT			0.13			0.0075
0	1 (0)	0 (0)		1 (0)	0 (0)	
1	20 (4)	1 (2)		21 (4)	0 (0)	
2	78 (15)	5 (8)		79 (16)	4 (6)	
3	363 (71)	48 (77)		360 (71)	51 (77)	
4	50 (10)	8 (13)		47 (9)	11 (17)	
pN			0.90			0.42
0	323 (64)	31 (52)		319 (64)	35 (53)	
1	133 (26)	18 (30)		132 (26)	19 (29)	
2	50 (10)	11 (18)		49 (10)	12 (18)	
Tumor differentiation			<0.0001			<0.001
High	51 (10)	5 (8)		52 (10)	4 (6)	
Moderate	406 (80)	25 (40)		399 (79)	32 (48)	
Low	48 (9)	29 (47)		50 (10)	27 (40)	
Mucinous	3 (1)	3 (5)		2 (0)	4 (6)	
Microsatellite instability			<0.0001			<0.0001
MSI	54 (11)	28 (47)		54 (11)	28 (47)	
MSS	435 (89)	32 (53)		435 (89)	32 (53)	
Tumor location			0.0064			0.0022
Right	193 (37)	42 (67)		188 (36)	47 (70)	
Left	182 (35)	9 (14)		180 (34)	11 (16)	
Rectum	138 (26)	10 (16)		140 (27)	8 (12)	
Synchronous	13 (2)	2 (3)		14 (3)	1 (1)	
Chemotherapy			0.11			0.61
Yes	58 (11)	9 (16)		55 (11)	12 (19)	
No	454 (89)	49 (84)		453 (89)	50 (81)	
Residual tumor			0.077			0.24
R0	414 (79)	44 (70)		410 (79)	48 (72)	
R1	24 (5)	1 (2)		23 (4)	2 (3)	
R2	87 (17)	18 (29)		88 (17)	17 (25)	

Numbers in parentheses indicate percentages. Of note, adjuvant chemotherapy was first offered as standard treatment in Norway from 1997 for patients with stage III colorectal cancer below 75 years of age. Tumor stage, pT and pN were determined according to TNM version 5. *P* values were calculated using Wilcoxon rank-sum test (2 independent variables) or Kruskal-Wallis H test (>2 independent variables) and indicate correlation between CDX2 expression and the indicated patient characteristic for Allred scores and DIA, respectively. Ungrouped Allred scores and continuous DIA scores were used as input for the statistical tests. Only overlapping cases with evaluable CDX2 expression were included in the analyses

*DIA* digital image analysis, *R*0 complete resection—no residual tumor, *R*1 microscopic residual cancer at the resection margin, *R*2 macroscopic or radiologic evidence of residual cancer

This study was endorsed by the Norwegian Data Protection Authority and the Regional Committee for Medical and Health Research Ethics, South-Eastern Norway (REK number 1.2005.1629). We obtained informed consent from all patients prior to enrollment and the research biobanks were constructed according to national legislation. The research was performed according to the Declaration of Helsinki.

### Immunohistochemistry

IHC assays were performed on 4 µm thick sections, using monoclonal antibodies, except for SOX9 (polyclonal).

The chromogenic stains were previously performed and visually evaluated according to the Allred method for β-catenin, E-cadherin, and SOX9 [12] and for CDX2 [13]. SOX2 staining was performed using the same protocol as for CDX2 (not previously published). Allred scores (ranging from 0 to 8) were calculated for each evaluable case and relevant cellular compartment by adding the estimated proportion of positive cells (score value ranging from 0 to 5; 0 = none, 1 ≤ 1%, 2 = 1–10%, 3 = 11–33%, 4 = 34–66%, and 5 = 67–100%) and the estimated intensity of staining (score value ranging from 0 to 3; 0 = negative, 1 = weak, 2 = intermediate, and 3 = strong) (Fig. S1). The fluorescent stains were performed on the last sections of the same TMA blocks and subsequent analyses included only two of the total four TMA blocks since the other two were exhausted. Cases with poor tumor preservation, loss of tissue, insufficient number of epithelial cells (typically <50), extensive tissue folding, or necrosis were excluded from the analyses. The number of evaluable and overlapping cases for each stain can be found in Table 2. Only overlapping cases with evaluable staining were used for comparative analyses.

**Table 2:** Number of evaluable and overlapping cases for Allred and DIA scoring of SOX9, CDX2, SOX2, β-catenin, and E-cadherin available for comparisons

Biomarker	Evaluable cases	Overlapping cases DIA/Allred	IHC staining method
CDX2 nucl (DIA)			4-plex IF mIHC
Min	0.03		
Max	6.5		
*n* (mean; sd)	373 (1.9 ± 1.4)	363	
CDX2 nucl (DIA)^a^			5-plex IF mIHC
Min	0.001		
Max	7.4		
*n* (mean; sd)	814 (1.1 ± 1.1)	589	
CDX2 nucl (Allred)			DAB chromogen + hematoxylin counterstain
Min	0		
Max	8		
*n* (mean; sd)	642 (7.4 ± 1.7)	363/589^b^	
SOX2 nucl (DIA)			4-plex IF mIHC
Min	0.006		
Max	23.6		
*n* (mean; sd)	373 (0.58 ± 1.88)	361	
SOX2 nucl (Allred)			DAB chromogen + hematoxylin counterstain
Min	0		
Max	8		
*n* (mean; sd)	645 (1.2 ± 2.6)	361	
SOX9 nucl (DIA)			3-plex IF mIHC
Min	0.13		
Max	5.3		
*n* (mean; sd)	181 (1.2 ± 1.0)	172	
SOX9 nucl (Allred)			DAB chromogen + hematoxylin counterstain
Min	0		
Max	8		
*n* (mean; sd)	761 (5.6 ± 1.5)	172	
β-catenin nucl (DIA)			3-plex IF mIHC
Min	0.045		
Max	34.5		
*n* (mean; sd)	380 (6.4 ± 5.8)	321	
β-catenin nucl (Allred)			DAB chromogen + hematoxylin counterstain
Min	0		
Max	8		
*n* (mean; sd)	637 (3.9 ± 2.0)	321	
β-catenin cyto (DIA)			3-plex IF mIHC
Min	0.1		
Max	21.2		
*n* (mean; sd)	380 (4.6 ± 3.7)	361	
β-catenin cyto (Allred)			DAB chromogen + hematoxylin counterstain
Min	0		
Max	8		
*n* (mean; sd)	722 (6.7 ± 1.3)	361	
E-cadherin cyto (DIA)			3-plex IF mIHC
Min	0.18		
Max	20.9		
*n* (mean; sd)	420 (5.1 ± 3.0)	386	
E-cadherin cyto (Allred)			DAB chromogen + hematoxylin counterstain
Min	0		
Max	8		
*n* (mean; sd)	720 (6.6 ± 0.9)	386	

For DIA, min, max, and mean are calculated from raw DIA scores

*DIA* digital image analysis, *nucl* nuclear, *cyto* cytoplasm, *mIHC* multiplex immunohistochemistry, *IF* immunofluorescence, *DAB* 3,3'-diaminobenzidine

^a^This stain was performed on TMA sections from a replicate TMA set and the data were only used for prognostic comparison between Allred scoring and DIA. All other stains were performed on sections with tissue cores from the same replicate TMA set and used for correlation analyses and visualizations. See Table S1 for an overview of the individual stains

^b^363 and 589 cases with evaluable CDX2 staining were overlapping with the 4-plex and the 5-plex IF mIHC stain, respectively

Indirect detection by fluorescence was based on the Opal^TM^ Multiplex IHC method (PerkinElmer/Akoya, USA), and performed on the Autostainer Link 48 system (Agilent/Dako, Denmark) with a PT link module to standardize the staining process. Deparaffinization, antigen retrieval, and antibody stripping were carried out for 20 min at 97 °C using the EnVision™ FLEX Target Retrieval Solution (3-in-1) pH 9 (Agilent/Dako), in 65 °C preheat mode. Subsequent staining was performed using the Opal^TM^ 4-Color Manual IHC Kit (PerkinElmer/Akoya, USA) according to the manufacturer’s recommendations. Signal amplification and covalent binding of fluorophore was achieved by using a tyramide signaling amplification reagent (included in the Opal kit) that is conjugated with a different fluorophore for each cycle [8]. Each fluorescent stain performed included markers for epithelial tissue and DAPI (described further below). Thus, in a 3-plex stain there is room for analysis of one biomarker, in a 4-plex there is room for two, and in a 5-plex there is room for three. A total of three 3-plex stains (for analysis of SOX9, β-catenin, and E-cadherin), one 4-plex stain (for analysis of CDX2 and SOX2), and one 5-plex stain (for analysis of CDX2 and two unpublished markers) were performed in the study (see also Table 2 for a list of all stains and Table S1 for an overview of the staining procedure for each multiplex stain and included biomarkers). Tissue samples were incubated for 30 min with the following primary antibodies: CDX2 (1:50, clone 88, Abcam, UK; detected by Opal 520 at 1:100), SOX2 (1:25, clone SP76, Cell Marque/Sigma-Aldrich, Germany; detected by Opal 570 at 1:100), SOX9 (1:500, Sigma-Aldrich; detected by Opal 570 at 1:100), E-cadherin (1:16000, clone 36, Becton Dickinson, USA; detected by Opal 570 at 1:100), and β-catenin (1:3000, clone 14, Becton Dickinson; detected by Opal 570 at 1:100). In the last cycle of antibody staining, the tissue was hybridized with a cocktail of epithelial markers to allow for complete and accurate epithelial segmentation by the DIA algorithm (anti-pan Cytokeratin (1:1500, clone C-11, Abcam) and anti-pan Cytokeratin Type I/II (1:1000, clone AE1/AE3, Thermo Fisher Scientific, USA); these were detected by Opal 670 at 1:100. For the 4- and 5-plex stains, anti-E-cadherin (1:16000, Clone 36, Becton Dickinson) was included in the epithelial antibody cocktail. Counterstaining was performed using DAPI (PerkinElmer/Akoya) according to the manufacturer’s protocol. Finally, the slides were mounted using ProLong Diamond Antifade Mountant (Invitrogen/Thermo Fisher Scientific). A separate single-plex stain was performed for each fluorophore to create spectral libraries for unmixing of individual spectral signatures in the multiplex. In addition, one slide was not probed with any fluorophore, thus providing the spectral signature of the tissue autofluorescence. The chosen concentration of antibodies was based on optimizing the staining specificity, signal intensity, and signal-to-noise level for both chromogenic DAB and fluorescence staining among control tissues embedded on a separate test TMA, including 42 primary colorectal cancer cases and six samples from normal colon mucosa (Fig. S2). Fluorescence signal intensities for all markers were balanced and kept within the recommended signal range for optimal spectral unmixing of fluorophores with the Vectra 3 system, at between 0 to about 30 counts with the UV lamp power set to 10%. In addition, a negative control experiment where the primary antibody was omitted was performed. To confirm that antibodies were properly stripped away or denatured between cycles [15], the following control experiment was performed for each antibody: after deparaffinization/antigen retrieval, sections were probed with the antibody and stained with its paired fluorophore. This was then followed by heat-treatment in the PT-link. A new round of staining was then performed, however, this time omitting any primary antibody and applying a different fluorophore after secondary antibody incubation. The tissue was imaged and analyzed to confirm that there was no signal above noise originating from the second fluorophore applied. Uniformity of staining was assessed visually and by scatterplots and Spearman’s correlation coefficients assessing the association between protein expression and sample age (Fig. S3).

Fluorescence-based detection of CDX2 was performed for two separate cocktails, one 4-plex with SOX2, and one 5-plex with two other markers on sections from a replicate TMA where samples from all blocks were available. Hence, the number of available cases for evaluation of CDX2 expression was much higher and facilitated comparison of prognostic value between the Allred method and DIA. 5-plex staining for CDX2 and other markers (data not shown) was performed manually using the Opal^TM^ 5-color Manual IHC Kit (PerkinElmer/Akoya) according to the manufacturer’s recommendations, except for deparaffinization, antigen retrieval and antibody stripping steps being performed in the PT link module, as described previously.

### Image acquisition and digital image analysis

Multispectral images were acquired at ×20 magnification using the Vectra 3.0 Automated Quantitative Pathology Imaging System, 200 slides (Vectra software version 3, PerkinElmer/Akoya). Standard settings were used for multispectral image acquisition.

Multispectral image analysis of multiplex IHC stains was performed using inForm Image Analysis Software (version 2.3, PerkinElmer/Akoya). A representative set of training images were first loaded and spectrally unmixed by using spectral libraries generated from the library stains for each fluorophore and the autofluorescence slide. Next, a machine learning algorithm was trained by user-specified tissue annotations aided by the signal from the epithelial markers to accurately segment tumor tissue versus stromal tissue and background, as well as individual cells using the nuclear DAPI signal. Optimization of the membrane segmentation algorithm for β-catenin and E-cadherin analysis was aided by the signal from the pan-cytokeratin staining. All images were reviewed after batch processing; normal glands, necrotic tissue, tissue folds, and other technical artefacts were excluded from further image analyses (Fig. S4). Protein expression was calculated in segmented tumor tissue as the mean signal intensity within the respective cellular compartment.

### Statistics

All statistical analyses were performed using RStudio version 1.1.463 (R version 3.3.2). Five-year overall survival plots with risk tables were generated according to the Kaplan–Meier method using the Survminer package (version 0.4.3). Survival curves were compared using the log rank test, and hazard ratios and 95% confidence intervals (CI) were estimated using univariable and multivariable Cox proportional hazards models. The overall survival time was defined from surgery to death from any cause. Follow-up was complete in the study period. Scatterplots were generated with the ggscatter function in the ggpubr package (version 0.1.6) using the Spearman method to calculate correlation coefficients and *P* values. Density distribution plots were generated using the ggdensity function in the ggpubr package (version 0.1.6). All *P* values were two-sided and derived from statistical tests with a significance level at 0.05.

## Results

### Reasonable concordance between visual and digital scoring, but digital analysis of membrane staining is challenging

**Fig. 1:**
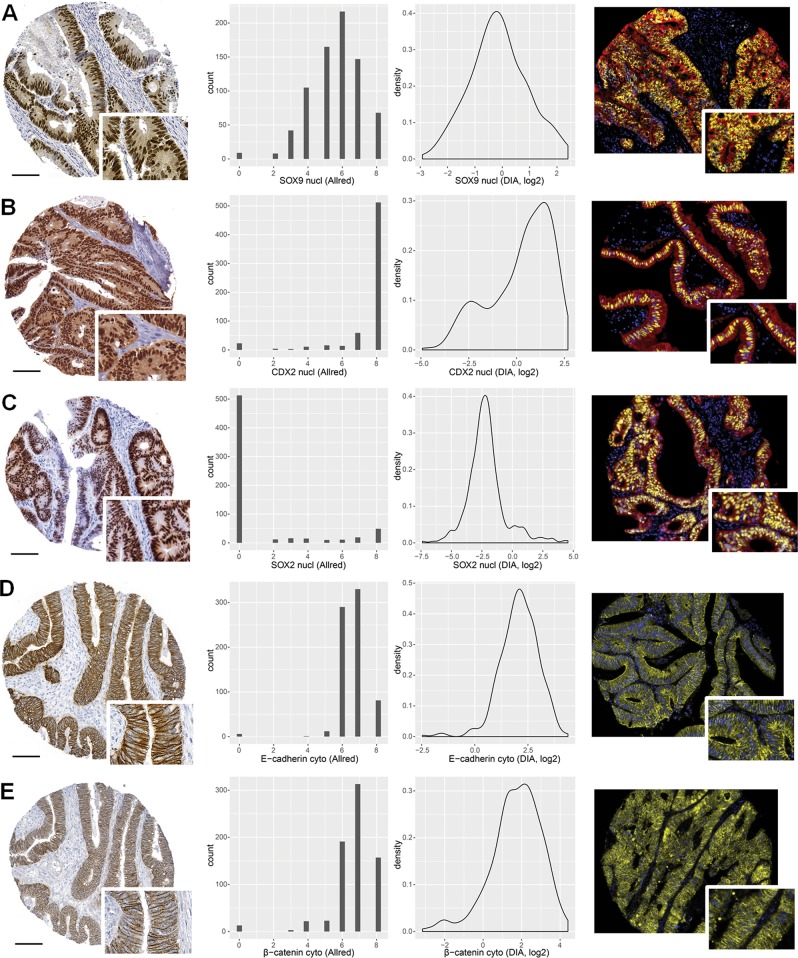
Representative images of chromogenic (left) and fluorescent (right) staining patterns for SOX9 (**a**), CDX2 (**b**), SOX2 (**c**), E-cadherin (**d**), and β-catenin (**e**). Distributions of Allred scores (middle-left column) and DIA (middle-right column) scores are shown in the middle columns for comparison; nuclear scores for SOX9, CDX2, and SOX2, and cytoplasmic scores for E-cadherin and β-catenin. DAPI staining is shown in blue, epithelial staining in red, and marker expression in yellow (**a–c**). Scale bar, 0.1 mm

The protein expression levels and patterns of CDX2, SOX2, SOX9, E-cadherin, and β-catenin were evaluated visually by singleplex chromogenic-based (DAB) IHC and by fluorescence-based mIHC. Both staining methods showed that all markers were expressed predominantly in epithelial cells (all but SOX2 were also expressed both in the normal mucosa and the cancer cells); CDX2, SOX2, and SOX9 were expressed predominantly in the cell nuclei, whereas E-cadherin was expressed in the cell membrane and cytoplasm, and β-catenin was expressed in all cellular compartments (Fig. 1).

**Fig. 2:**
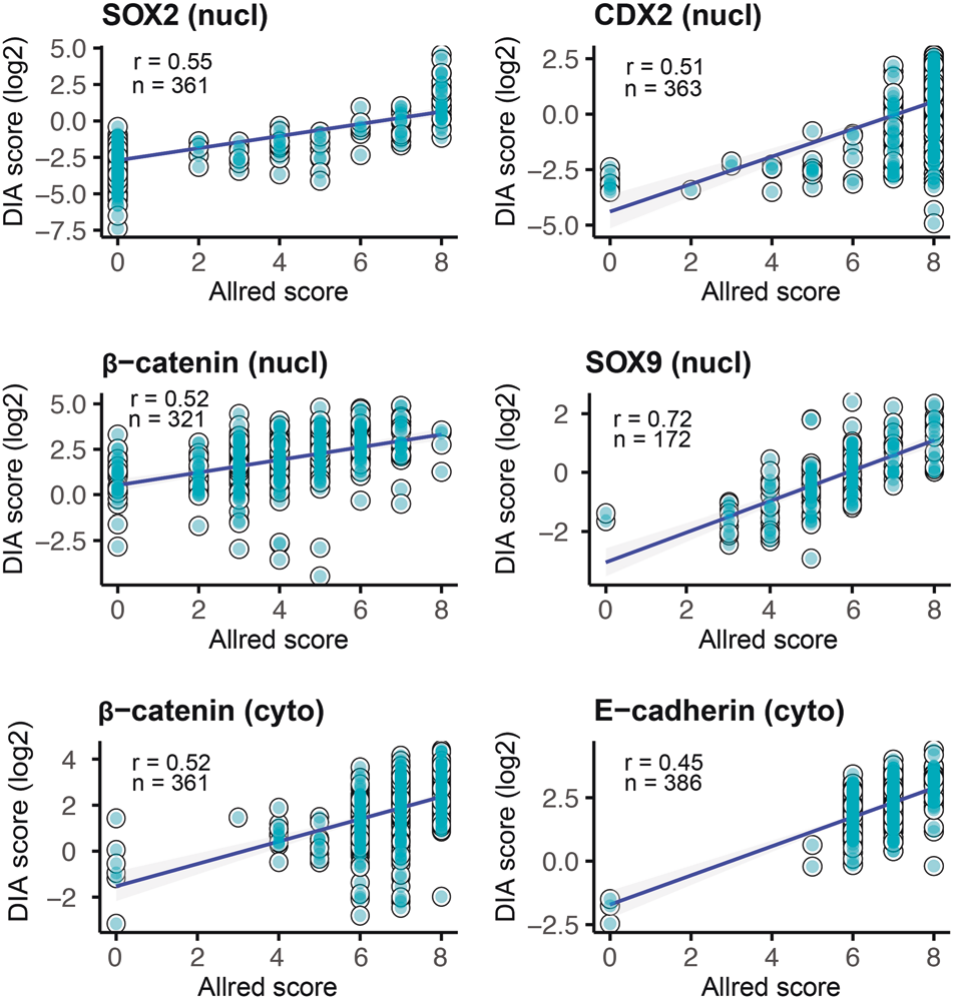
Correlation between Allred and DIA scores for CDX2, SOX2, SOX9, E-cadherin, and β-catenin. Correlation coefficients were calculated using the Spearman’s rho method. DIA scores were log_2_ transformed for visualization. nucl nucleus, cyto cytoplasm, mem membrane, DIA digital image analysis

**Fig. 3:**
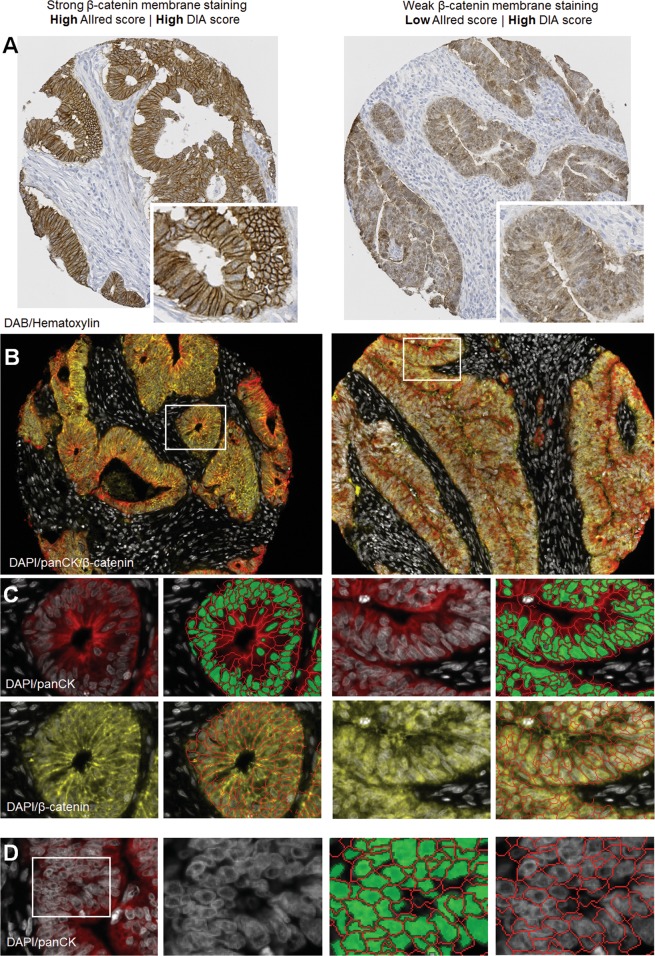
Challenges with digital analysis of membrane staining. Illustration of tumor cores displaying strong (**a**, left side) and weak (**a**, right side) membrane staining for β-catenin, visualized by the chromogenic substrate DAB. The same staining pattern is seen by fluorescent labeling (**b**); pan-cytokeratin (panCK) is shown in red, DAPI in white, and β-catenin in yellow. Digital analysis of β-catenin membrane staining in the epithelium based on nuclear segmentation (green segments) and membrane segmentation (red lines) aided by nuclear DAPI staining and panCK membrane staining (**c**). Membrane regions are fairly well segmented, and in the example on the left side, these regions pick up strong β-catenin staining correctly. However, in the example on the right side, the segmented membrane region is primarily picking up diffuse cytoplasmic β-catenin staining. Illustration of membrane segmentation in densely nucleated tissue areas showing how signal originating from the nuclei may be picked up in the segmented membrane region (**d**)

The chromogenic stains were scored visually within the epithelial compartment with discrete values from 0 to 8, while the fluorescent stains were scored digitally on a continuous scale within the epithelial compartment. The distributions of nuclear and cytoplasmic scores were similar between the two methods (Fig. 1, middle) and they showed a reasonable concordance considering the inherent differences in scoring methodology (Spearman’s rho test, correlation coefficients (*r*) from 0.45 to 0.72; Fig. 2), but analysis of membrane staining showed a poor concordance (Spearman’s rho test, *r* = 0.095 for β-catenin and *r* = 0.39 for E-cadherin, Fig. S5). This can in part be explained by challenges with the membrane segmentation algorithm (Fig. 3a–d). Hence, further comparisons for analysis of membrane staining were not performed.

### Fluorescence-based IHC combined with DIA captures variation in protein expression within Allred scoring groups and improves differentiation among cases

**Fig. 4:**
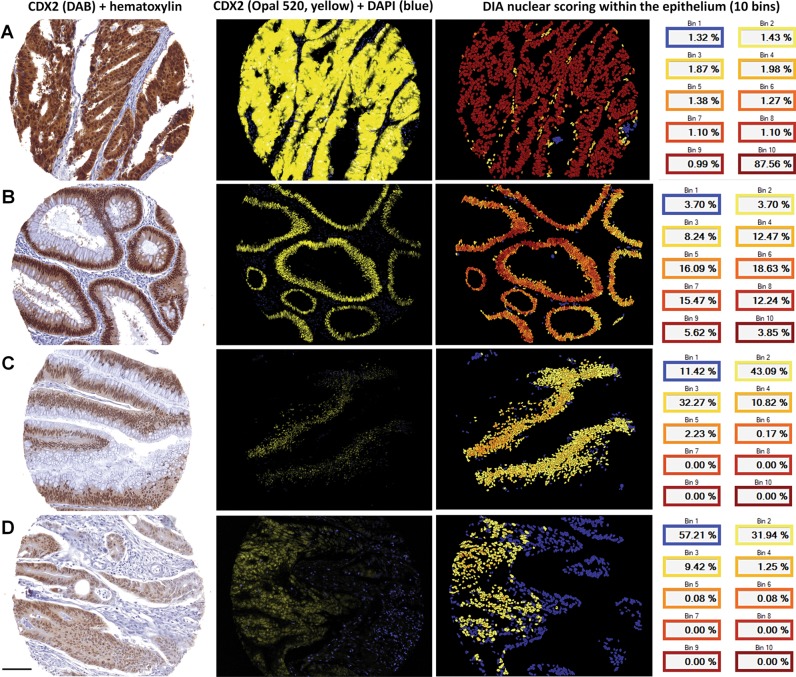
Strongly and weakly stained cases for CDX2 are better separated by fluorescence-based IHC, which has a higher data resolution compared with chromogenic detection using 3,3′-diaminobenzidine (DAB). Three cases illustrating the large variation in CDX2 protein expression among cases with Allred score 8 (**a–c**). Chromogenic staining with DAB is prone to signal saturation, whereas fluorophores have a much larger linear dynamic signal range enabling DIA to more accurately detect and quantify differential protein expression on a continuous scale. Example of a discrepant case with CDX2 Allred score 8 and DIA score in the lowest quartile showing how cases with clearly reduced protein expression can be scored as strong because the dynamic signal range of DAB is not sufficient to differentiate both the weak and the strong cases, leading to some of the weaker cases being stained too strong to be readily separated by visual analysis (**d**). Single-cell analysis (right image column) furthermore enables a more accurate and objective scoring of cases with large variation in protein expression between individual cells, here illustrated by the gradual difference in CDX2 expression from left to right on the histospot which is less noticeable for the DAB stain. CDX2 signal intensities were binned into 10th percentiles on a cell-by-cell basis. Bin1 corresponds to the lower percentile (blue colour) and Bin10 (dark red colour) to the higher percentile. Fluorescent images are scaled relative to each other; hence CDX2 staining in A appears oversaturated due to the relatively much higher protein expression in this sample. Scale bar, 0.1 mm

In general, we observed a large variation in protein expression scores from the fluorescently labeled and digitally analyzed samples within the Allred scoring groups. To illustrate this variation we selected three samples that were scored into the highest Allred category (Allred = 8) for CDX2 expression, but which had large differences in rank (and absolute score) when analyzed by fluorescence and DIA. The distribution of Allred scores were somewhat shifted toward the higher values as compared with the DIA scores (Figs. 1, 2). We also observed that minor differences between samples stained with DAB could translate to much larger differences when the samples were stained with fluorescent probes (Fig. 4a–c). DIA analysis at the single cell level classifying CDX2 protein expression into ten bins showed that the protein expression differed substantially between these cases, although this was not evident by DAB-staining and visual analysis. DIA accurately quantified the fluorescence signal from each individual cell and calculated the average signal per case and was thus able to objectively measure the average protein expression also in samples with heterogeneous staining patterns (Fig. 4d), which are difficult to assess by visual analysis.

### Multiplex IHC combined with DIA improves detection of histopathological and biological relationships

**Fig. 5:**
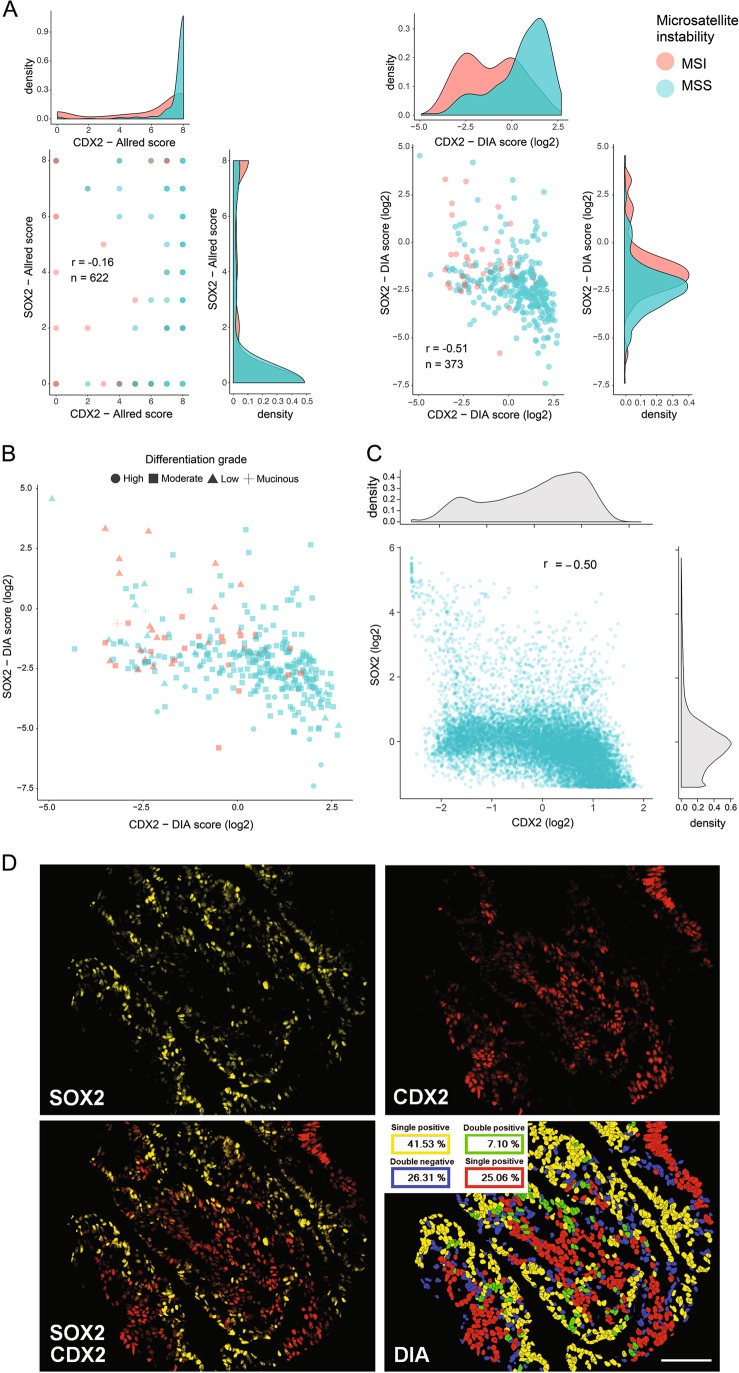
Illustrations of clinicopathological and biological relationships analyzed by chromogenic and fluorescent IHC. Scatterplots for Allred and DIA scoring separately show the inverse relationship between CDX2 and SOX2 and their association with microsatellite instability (MSI). Accompanying density plots show the probability distribution of each variable (**a**). Relationship between CDX2, SOX2, MSI-status, and differentiation grade; tumors with low CDX2 expression tend to have high SOX2 expression, show MSI and have a low differentiation grade (**b**). Single-cell analysis of CDX2 and SOX2 (C/D). Distribution of CDX2 and SOX2 scores at the single-cell level (**c**). Scores were calculated as the mean fluorescent intensity within individual cell nuclei. The plot was downsampled by randomly selecting 10,000 cells for analysis to facilitate visualization. Illustrative example of a nearly mutually exclusive relationship between CDX2 and SOX2 at the single-cell level (**d**). Thresholding was performed automatically within the Inform Software for visualization. Correlation coefficients were calculated using the Spearman’s rho method. DIA scores were log_2_ transformed for visualization. Colocalization analysis was performed by inForm Image Analysis Software Version 2.3. Scale bar, 0.1 mm

By analyzing both serial stains and the multiplex staining of CDX2 and SOX2 we confirmed their inverse relationship in colorectal cancer [16]; however, the inverse correlation was considerably stronger for DIA (Spearman’s rho test, Allred *r* = −0.16; DIA *r* = −0.51; *n* = 357; Fig. 5a). Furthermore, the DIA method also detected a stronger association between loss of CDX2 and MSI, and this result could be effectively visualized by the denser distribution of MSI tumors with low CDX2-expression (Spearman’s rho test, Allred *r* = 0.26, DIA *r* = 0.31; *n* = 343, Fig. 5a). Similarly, the correlation between SOX2 and MSI was stronger for DIA as well (Spearman’s rho test, Allred *r* = 0.088, DIA *r* = 0.26; *n* = 341, Fig. 5a). The correlation between loss of CDX2 and a low differentiation grade was similar for the two methods (Spearman’s rho test, Allred *r* = 0.22; DIA *r* = 0.18; *n* = 349). Overall, the correlations among markers and with clinicopathological variables were stronger for the digital analysis as compared with the visual analysis (Fig. S6). Continuous data, having a higher resolution, are particularly suited to visualize these biological relationships, as illustrated in Fig. 5b. We performed single-cell analysis of CDX2/SOX2 colocalization for the multiplex staining and found a similarly strong inverse relationship between the two markers (Fig. 5c). Interestingly, a small subset of the tumors showed a nearly mutually exclusive expression on the single cell-level (Fig. 5d).

### DIA recapitulates prognostic associations for the CDX2 biomarker

**Fig. 6:**
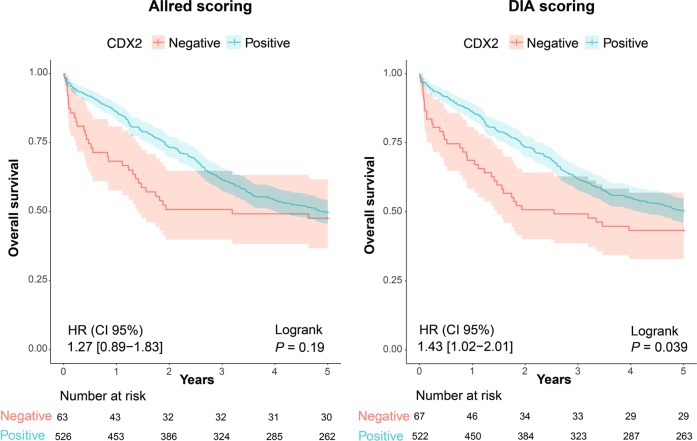
Comparison of prognostic assessments of the biomarker CDX2 using Allred and DIA scoring in primary colorectal cancers from stage I to IV employing a predetermined cut-off for CDX2-positivity at the 11th percentile [16]. Thresholding was performed using all cases with information on CDX2. Survival analysis includes only cases with both Allred and DIA scoring data for CDX2. Univariable Cox regression was used to generate hazard ratios (HR) and 95% confidence intervals (CI)

To evaluate potential differences in predictive performance, the two analytical approaches were compared with respect to their ability to detect well known prognostic relationships for the biomarker CDX2 [13, 17, 18]. A predetermined cutoff for CDX2-positivity at the 11th percentile was used to reduce confirmation bias and was originally set near the inflection point for the bimodal distribution using the Binarization Across multiple SCales algorithm [13]. We confirmed that the fluorescence-based CDX2 protein expression data showed a similar bimodal distribution (Fig. 1b). Kaplan–Meier analysis of 5-year overall survival showed that DIA confirmed the association between a low CDX2 expression and a poor prognosis (Allred: HR 1.27, 95% CI 0.89–1.83, *P* = 0.19; DIA: HR 1.43, CI 1.02–2.01, *P* = 0.039; *n* = 589; Fig. 6; patient characteristics Table 1), as well as recapitulating the strong prognostic value of CDX2 in stage IV colorectal cancer (Fig. S7) [13]. Results were also similar in multivariable analyses including the covariates age, gender, tumor stage, MSI, and differentiation grade (Allred: HR 1.54, 95% CI 1.01–2.38, *P* = 0.047; DIA: HR 1.81, 95% CI 1.17–2.80, *P* = 0.0072; *n* = 530).

## Discussion

### Overall a good correlation between visual and digital biomarker analyses

Our study supports fluorescence-based mIHC combined with DIA using machine learning as a good method to quantify biomarker expression. We obtained reasonable correlations for nuclear and cytoplasmic staining when comparing chromogenic singleplex IHC with fluorescent mIHC results for five known colorectal cancer markers (CDX2, SOX2, SOX9, E-cadherin, and β-catenin), considering that the scoring methodologies are inherently different and that the tissue sections used to compare these methodologies were not neighboring sections in the TMA. Similar correlations between the two scoring methods have been reported for several cancer types [19–25].

### The nature of the staining and scoring methods explains inconsistencies

Nonetheless, scores from one method are not directly translatable to the other. These differences between methods can in part be attributed to technical issues. First, the fact that the tissue sections used for chromogenic and fluorescent staining were not adjacent reduces the accuracy of the comparison between the methods. Also, loss of tissue, detachment during processing, and staining of “exhausted” paraffin blocks limited the comparisons. For downstream survival analyses with respect to the biomarker CDX2 we stained a replicate TMA set to increase the number of samples analyzed by fluorescence. Even though the samples stained by DAB and fluorescence were from different areas of the same donor block, the survival analyses remained highly similar. Further, it is important to keep in mind the inherent differences between DAB-based visual (Allred) scoring and fluorescence-based digital analyses. Inconsistencies between data obtained with the two scoring approaches are partly due to DIA being a more quantitative method that yields continuous values, while visual assessment according to the Allred score produces discrete values on a nonlinear and discontinuous scale from 0 to 8. For example, the Allred scores 4, 5, and 6 can be ambiguous since different staining patterns can underlie these scores, typically biasing the evaluation of results toward high scores and masking variability within tumor samples. Here, DIA offers a simpler and more objective approach to accurately measure the protein expression in the tissue. Saturation of DAB signal leading to compression and right-shifting of the data distribution is an additional likely explanation for some of the observed discrepancies with DIA scores.

Of note, the ‘optimal’ scoring approach may vary from biomarker to biomarker. Some proteins may exert their strongest influence on biology by number, with increasing amounts of protein being related to some cellular or tumor phenotype. Other biomarkers may be better described by the number of cells expressing the protein. The Allred score is based on categorization of each of these parameters prior to summation, while the scores provided through the current DIA algorithm are intrinsically based on both of these measures by analyzing the mean score across all tumor cells. This scoring method for direct comparison of DIA with Allred was chosen as it does not require any definition of threshold for biomarker positivity and is thus robust. However, with the cell-by-cell data obtained through DIA, more complex scoring schemes are straightforward to develop. For instance, cells can readily be categorized based on expression levels and analyzed for colocalization with other markers, as illustrated for CDX2 and SOX2.

Furthermore, visual scoring is performed with dichotomization in mind, meaning that uncertain “strong” or “weak” cases are typically given a score closer to the middle (classified as “moderate”), to keep the “strong” and “weak” categories robust. Importantly, chromogenic detection has a narrow linear dynamic range [6] and reaches saturation fast, thus being prone to compressing “moderate” and “strong” signals as compared with fluorescence-based detection. Accordingly, fluorescence-based IHC provides the basis for more accurate quantification of protein expression for cases at the high end of the spectrum, particularly for proteins with a large expression range.

### Scoring of membrane staining: a challenge for the digital analysis

Evaluation of membrane staining is relevant for many clinically important biomarkers, such as β-catenin, E-cadherin, and HER2. Unfortunately, the digital scoring of membrane staining correlated poorly with the visual analysis, which was better at discerning cytoplasmic and nuclear staining from specific membrane staining. This observation might have several explanations, including inherent limitations of the machine learning algorithm to segment cell–cell borders consistently across cases with different morphologies and technical issues, despite thorough optimization of segmentation parameters. Also, the specificity of the marker used for guiding the membrane segmentation, inherent limitations set by image resolution, various physical characteristics of the tissue sections where individual cells/nuclei, and membranes are often not distinguishable, as well as general difficulties in differentiating between diffuse cytoplasmic staining and specific membrane staining at cell–cell borders, are other important factors that can explain the discrepancy between the scoring methods. The staining pattern of the biomarker is inherently important for how well the methods compare; β-catenin, due to its potential presence in any cellular compartment is more sensitive to inaccuracies in compartment-based scoring, when compared with scoring of a biomarker that is more or less exclusively found in one cellular compartment. That said, it is possible that a more specific membrane marker and alternative software packages and machine learning tools could mitigate some of these limitations.

### Digital analysis facilitates biomarker combination assessments and confirms the prognostic value of CDX2

We found a strong inverse relationship between the expression levels of CDX2 and SOX2 in colorectal cancer, confirming a previous study by Lundberg et al. [16]. This correlation is well documented in the gastric setting, namely in intestinal metaplasia [26–28]. We also confirmed previous observations showing that low CDX2 expression is associated with the MSI phenotype [13, 18]. We further observed that tumors with low CDX2 expression typically have high SOX2 expression and are poorly differentiated, and demonstrated that identification and visualization of these clinicopathological and biological relationships can be substantially improved by the higher and linear data resolution obtained with a multiplex fluorescence-based IHC approach combined with DIA, as well as by the ability to perform serial stains on the same tissue section. The value of fluorescence-based mIHC combined with DIA technology for colocalization analyses [29] has been well demonstrated in immunoprofiling studies [9, 19, 30–32], and we illustrate the feasibility of such analyses also for assessing the relationship between important tumor differentiation markers such as CDX2 and SOX2.

We also show that mIHC combined with DIA is an efficient approach to assess the prognostic value of CDX2 protein expression, highlighting the potential clinical utility of this technology to assess nuclear and cytoplasmic markers in a more standardized fashion, in line with results in esophageal cancer [33], breast cancer [34], and colorectal cancer [35]. The Wistuba lab recently reviewed multiplex staining and DIA platforms, concluding with their utility and advantages for translational research and clinical applications [36], and a recent systematic review and meta-analysis of biomarker modalities for predicting response to PD-1/PD-L1 checkpoint blockade concluded that mIHC has improved diagnostic performance as compared with conventional PD-L1 IHC, tumor mutational burden, and gene expression profiling [37].

## Conclusions

In conclusion, fluorescence-based mIHC combined with DIA is a reliable and efficient method to quantify biomarker protein expression in TMAs and to detect clinicopathological and biological relationships, although robust analysis of membrane staining remains a challenge. Our results advocate the use of mIHC and DIA for research and clinical applications, here successfully shown for the colorectal cancer biomarker CDX2.

## Supplementary information

Supplementary material

## References

[1] Wan WH, Fortuna MB, Furmanski P (1987). A rapid and efficient method for testing immunohistochemical reactivity of monoclonal antibodies against multiple tissue samples simultaneously. J Immunol Methods..

[2] Kononen J, Bubendorf L, Kallioniemi A, Barlund M, Schraml P, Leighton S (1998). Tissue microarrays for high-throughput molecular profiling of tumor specimens. Nat Med.

[3] Aeffner F, Wilson K, Martin NT, Black JC, Hendriks CLL, Bolon B (2017). The gold standard paradox in digital image analysis: manual versus automated scoring as ground truth. Arch Pathol Lab Med..

[4] Allred DC, Harvey JM, Berardo M, Clark GM (1998). Prognostic and predictive factors in breast cancer by immunohistochemical analysis. Mod Pathol..

[5] McCarty KS, Szabo E, Flowers JL, Cox EB, Leight GS, Miller L (1986). Use of a monoclonal anti-estrogen receptor antibody in the immunohistochemical evaluation of human tumors. Cancer Res..

[6] Rimm DL (2006). What brown cannot do for you. Nat Biotechnol.

[7] Huang W, Hennrick K, Drew S (2013). A colorful future of quantitative pathology: validation of Vectra technology using chromogenic multiplexed immunohistochemistry and prostate tissue microarrays. Hum Pathol..

[8] Stack EC, Wang C, Roman KA, Hoyt CC (2014). Multiplexed immunohistochemistry, imaging, and quantitation: a review, with an assessment of tyramide signal amplification, multispectral imaging and multiplex analysis. Methods..

[9] Gorris MAJ, Halilovic A, Rabold K, van Duffelen A, Wickramasinghe IN, Verweij D (2018). Eight-color multiplex immunohistochemistry for simultaneous detection of multiple immune checkpoint molecules within the tumor microenvironment. J Immunol..

[10] Ghaznavi F, Evans A, Madabhushi A, Feldman M (2013). Digital imaging in pathology: whole-slide imaging and beyond. Annu Rev Pathol..

[11] Levenson RM, Borowsky AD, Angelo M (2015). Immunohistochemistry and mass spectrometry for highly multiplexed cellular molecular imaging. Lab Investig.

[12] Bruun J, Kolberg M, Nesland JM, Svindland A, Nesbakken A, Lothe RA (2014). Prognostic significance of beta-Catenin, E-Cadherin, and SOX9 in colorectal cancer: results from a large population-representative series. Front Oncol..

[13] Bruun Jarle, Sveen Anita, Barros Rita, Eide Peter W., Eilertsen Ina, Kolberg Matthias, Pellinen Teijo, David Leonor, Svindland Aud, Kallioniemi Olli, Guren Marianne G., Nesbakken Arild, Almeida Raquel, Lothe Ragnhild A. (2018). Prognostic, predictive, and pharmacogenomic assessments of CDX2 refine stratification of colorectal cancer. Molecular Oncology.

[14] Merok MA, Ahlquist T, Royrvik EC, Tufteland KF, Hektoen M, Sjo OH (2013). Microsatellite instability has a positive prognostic impact on stage II colorectal cancer after complete resection: results from a large, consecutive Norwegian series. Ann Oncol..

[15] Blom S, Paavolainen L, Bychkov D, Turkki R, Maki-Teeri P, Hemmes A (2017). Systems pathology by multiplexed immunohistochemistry and whole-slide digital image analysis. Sci Rep.

[16] Lundberg IV, Edin S, Eklof V, Oberg A, Palmqvist R, Wikberg ML (2016). SOX2 expression is associated with a cancer stem cell state and down-regulation of CDX2 in colorectal cancer. BMC Cancer..

[17] Dalerba P, Sahoo D, Paik S, Guo X, Yothers G, Song N (2016). CDX2 as a prognostic biomarker in Stage II and Stage III colon cancer. N Engl J Med.

[18] Bae JM, Lee TH, Cho NY, Kim TY, Kang GH (2015). Loss of CDX2 expression is associated with poor prognosis in colorectal cancer patients. World J Gastroenterol..

[19] Mezheyeuski A, Bergsland CH, Backman M, Djureinovic D, Sjoblom T, Bruun J (2018). Multispectral imaging for quantitative and compartment-specific immune infiltrates reveals distinct immune profiles that classify lung cancer patients. J Pathol..

[20] Fiore C, Bailey D, Conlon N, Wu X, Martin N, Fiorentino M (2012). Utility of multispectral imaging in automated quantitative scoring of immunohistochemistry. J Clin Pathol..

[21] Desmeules P, Hovington H, Nguile-Makao M, Leger C, Caron A, Lacombe L (2015). Comparison of digital image analysis and visual scoring of KI-67 in prostate cancer prognosis after prostatectomy. Diagn Pathol..

[22] Rizzardi AE, Johnson AT, Vogel RI, Pambuccian SE, Henriksen J, Skubitz AP (2012). Quantitative comparison of immunohistochemical staining measured by digital image analysis versus pathologist visual scoring. Diagn Pathol..

[23] Turbin DA, Leung S, Cheang MC, Kennecke HA, Montgomery KD, McKinney S (2008). Automated quantitative analysis of estrogen receptor expression in breast carcinoma does not differ from expert pathologist scoring: a tissue microarray study of 3,484 cases. Breast Cancer Res Treat..

[24] Koopman T, Buikema HJ, Hollema H, de Bock GH, van der Vegt B (2018). Digital image analysis of Ki67 proliferation index in breast cancer using virtual dual staining on whole tissue sections: clinical validation and inter-platform agreement. Breast Cancer Res Treat..

[25] Ong CW, Kim LG, Kong HH, Low LY, Wang TT, Supriya S (2010). Computer-assisted pathological immunohistochemistry scoring is more time-effective than conventional scoring, but provides no analytical advantage. Histopathology..

[26] Niu H, Jia Y, Li T, Su B (2017). SOX2 inhibition promotes promoter demethylation of CDX2 to facilitate gastric intestinal metaplasia. Dig Dis Sci..

[27] Camilo V, Garrido M, Valente P, Ricardo S, Amaral AL, Barros R (2015). Differentiation reprogramming in gastric intestinal metaplasia and dysplasia: role of SOX2 and CDX2. Histopathology..

[28] Tsukamoto T, Inada K, Tanaka H, Mizoshita T, Mihara M, Ushijima T (2004). Down-regulation of a gastric transcription factor, Sox2, and ectopic expression of intestinal homeobox genes, Cdx1 and Cdx2: inverse correlation during progression from gastric/intestinal-mixed to complete intestinal metaplasia. J Cancer Res Clin Oncol..

[29] Bauman TM, Ricke EA, Drew SA, Huang W, Ricke WA. Quantitation of protein expression and co-localization using multiplexed immuno-histochemical staining and multispectral imaging. J Vis Exp. 2016;(110):53837. 10.3791/53837 PMC4913774 27167094

[30] Schalper KA, Carvajal-Hausdorf D, McLaughlin J, Altan M, Velcheti V, Gaule P (2017). Differential expression and significance of PD-L1, IDO-1, and B7-H4 in human lung cancer. Clin Cancer Res..

[31] Parra ER, Uraoka N, Jiang M, Cook P, Gibbons D, Forget MA (2017). Validation of multiplex immunofluorescence panels using multispectral microscopy for immune-profiling of formalin-fixed and paraffin-embedded human tumor tissues. Sci Rep.

[32] Ying L, Yan F, Meng Q, Yuan X, Yu L, Williams BRG (2017). Understanding immune phenotypes in human gastric disease tissues by multiplexed immunohistochemistry. J Transl Med..

[33] Feuchtinger A, Stiehler T, Jutting U, Marjanovic G, Luber B, Langer R (2015). Image analysis of immunohistochemistry is superior to visual scoring as shown for patient outcome of esophageal adenocarcinoma. Histochem Cell Biol..

[34] Stalhammar G, Fuentes Martinez N, Lippert M, Tobin NP, Molholm I, Kis L (2016). Digital image analysis outperforms manual biomarker assessment in breast cancer. Mod Pathol..

[35] Nolte S, Zlobec I, Lugli A, Hohenberger W, Croner R, Merkel S (2017). Construction and analysis of tissue microarrays in the era of digital pathology: a pilot study targeting CDX1 and CDX2 in a colon cancer cohort of 612 patients. J Pathol Clin Res..

[36] Parra Edwin, Francisco-Cruz Alejandro, Wistuba Ignacio (2019). State-of-the-Art of Profiling Immune Contexture in the Era of Multiplexed Staining and Digital Analysis to Study Paraffin Tumor Tissues. Cancers.

[37] Lu Steve, Stein Julie E., Rimm David L., Wang Daphne W., Bell J. Michael, Johnson Douglas B., Sosman Jeffrey A., Schalper Kurt A., Anders Robert A., Wang Hao, Hoyt Clifford, Pardoll Drew M., Danilova Ludmila, Taube Janis M. (2019). Comparison of Biomarker Modalities for Predicting Response to PD-1/PD-L1 Checkpoint Blockade. JAMA Oncology.

