# Supplementary material for: Digital image analysis of multiplex fluorescence IHC in colorectal cancer recognizes the prognostic value of CDX2 and its negative correlation with SOX2
Source: Lab Invest. 2019 Oct 22;100(1):120–34. doi: 10.1038/s41374-019-0336-4 (PMC6917572; doi:10.1038/s41374-019-0336-4)
Supplement: Supplementary file 1 — Supplementary material [file 41374_2019_336_MOESM1_ESM.pdf]

**Digital image analysis of multiplex fluorescence IHC in colorectal cancer recognizes the prognostic value of CDX2 and its negative correlation with SOX2**


Nair Lopes[1,2,3,4]*, Christian Holst Bergsland[1,4,5]*, Merete Bjørnslett[1,4], Teijo Pellinen[4,6], Aud Svindland[4,5], Arild Nesbakken[4,5,7], Raquel Almeida [2,8,9], Ragnhild A. Lothe[1,4,5], Leonor David[2,3,8], Jarle Bruun[1,4#]

1 – Department of Molecular Oncology, Institute for Cancer Research, Oslo University Hospital, Norway

2 – i3S – Institute for Research and Innovation in Health, University of Porto, Porto, Portugal

3 – IPATIMUP – Institute of Molecular Pathology and Immunology of the University of Porto, Porto, Portugal

4 – K.G. Jebsen Colorectal Cancer Research Centre, Division of Cancer Medicine, Oslo University Hospital, Norway

5 – Institute for Clinical Medicine, Faculty of Medicine, University of Oslo, Norway

6 – Institute for Molecular Medicine Finland (FIMM), University of Helsinki, Finland

7 – Department of Gastrointestinal Surgery, Oslo University Hospital, Oslo, Norway

8 – Faculty of Medicine, University of Porto, Porto, Portugal

9 –Department of Biology, Faculty of Sciences, University of Porto, Porto, Portugal

* both authors contributed equally to this work


Running title: Digital image analysis in colorectal cancer


# corresponding author: Jarle Bruun, Department of Molecular Oncology, Institute for Cancer Research, Oslo University Hospital HE – Norwegian Radium Hospital, Montebello P. O. Box 4953 Nydalen, NO-0424 Oslo, Norway. jarle.bruun@rr-research.no

## Supplementary figures and tables with legends

Allred score 0
(proportion of positive cells = 0,
intensity of staining = 0)

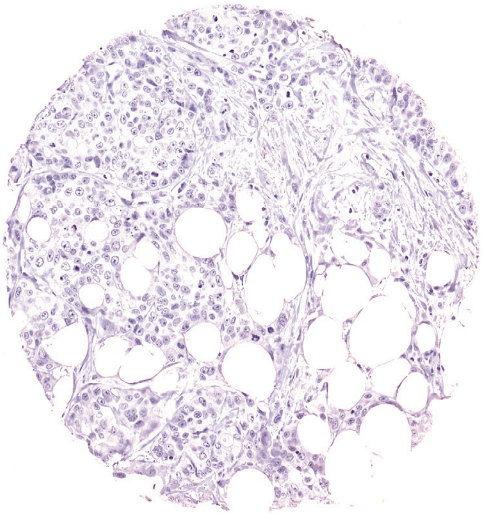

Allred score 4
(proportion of positive cells = 3,
intensity of staining = 1)

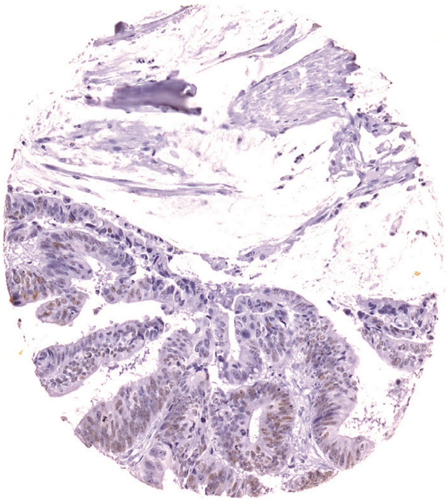

Allred score 6
(proportion of positive cells = 5,
intensity of staining = 1)

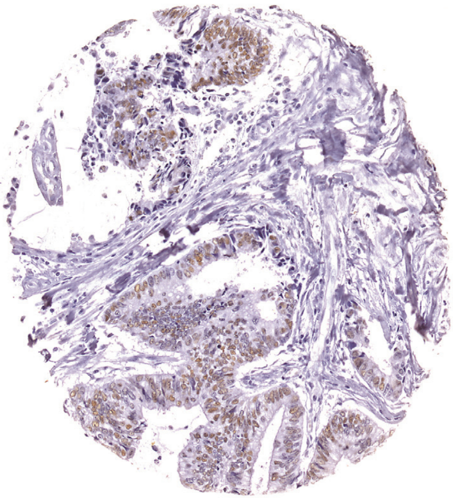

Allred score 7
(proportion of positive cells = 5,
intensity of staining = 2)

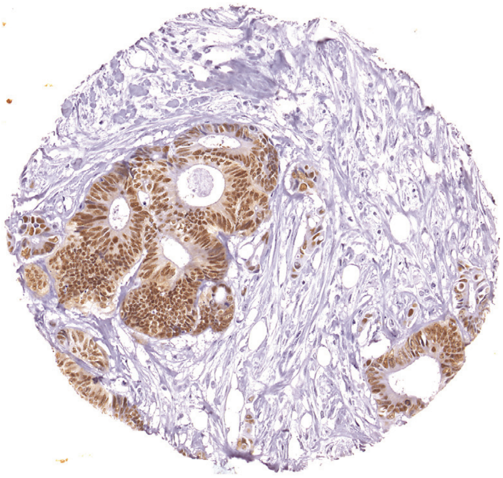

Allred score 8
(proportion of positive cells = 5, intensity of staining = 3)

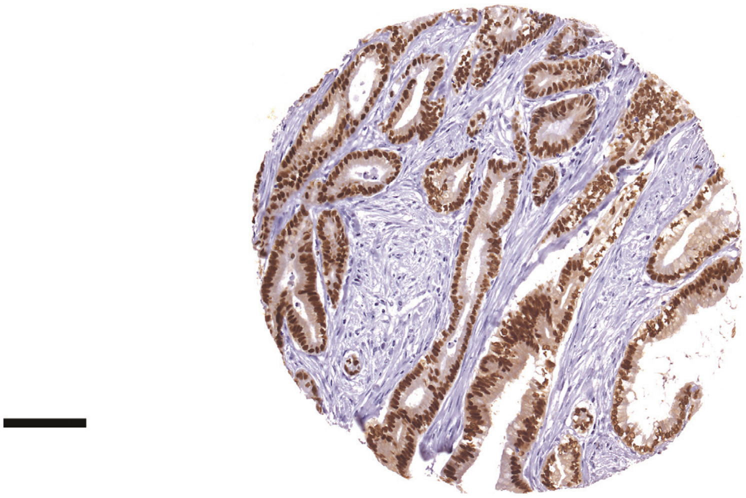

**Figure S1 |** Illustration of Allred scoring for CDX2 nuclear protein expression in colorectal cancer epithelium. Five tissue spots with common Allred scores are shown to illustrate the overall chromogenic DAB staining variation in the cohort. Of note, visual scoring is prone to difficulties with assessing accurately the percentages of positive cells which lead to variation in scoring of borderline cases. The prevalence of different cells showing various degrees of staining intensity within the evaluated tissue further complicates visual scoring of protein staining into defined categories. Scale marker, 0.1 mm.

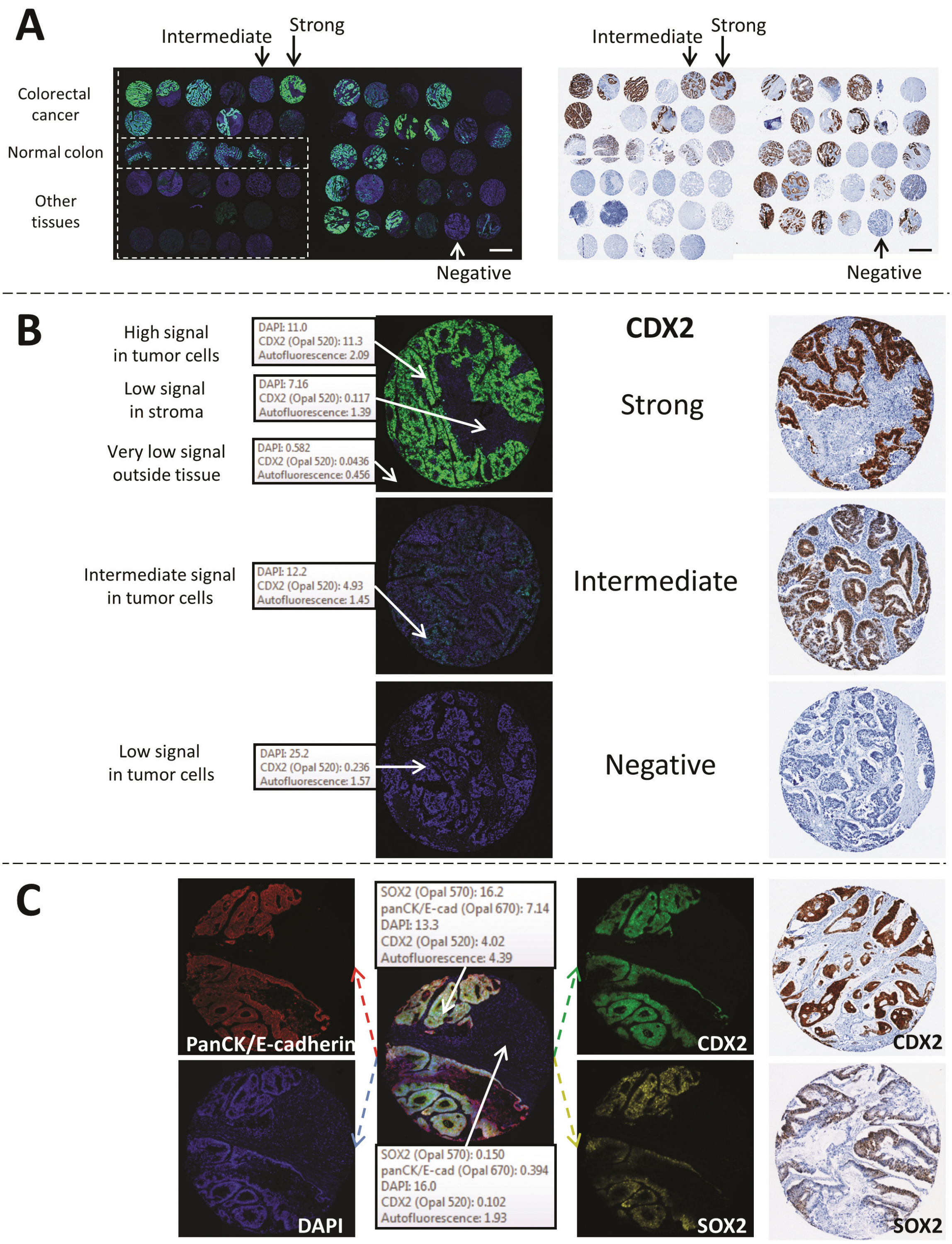


**Figure S2 |** Illustration of optimization of fluorescence-based CDX2-staining using test tissue microarrays (TMA). The test-TMA includes mainly colorectal cancer cases (42 colorectal cancers, 6 normal colon and 17 other control tissues, including normal tissue samples from lymph nodes, nerve tissues, kidney, heart and testis; 1 mm diameter core size). First, a fluorescence-based stain (CDX2; green, DAPI; blue) is directly compared to DAB-based chromogenic staining (CDX2; brown, hematoxylin; blue) to verify that staining patterns are

consistent and in accordance with *a priori* knowledge (A). Samples are then multispectrally imaged with the Vectra 3 platform and assessed using the Inform image analysis software; three examples are shown here (B). This is done to ensure that signal intensities are within the recommended range and to ensure that the signal-to-noise ratio is high (more than tenfold is recommended). Finally, multiplex staining is performed on the test-TMA, images are unmixed using Inform and signal intensities are verified (C). This is done to confirm that the multiplex staining is in line with what was observed when the test-TMA was stained against only one marker and that signals ranges are adequate for spectral unmixing. A mixture of panCK (pan-cytokeratin) and E-cadherin antibodies were used as epithelial markers for segmentation of tumor tissue in the digital image analysis of the 4-plex. Images of the same tissue core stained against CDX2 and SOX2 by DAB are shown for comparison. Scale bar, 1 mm.

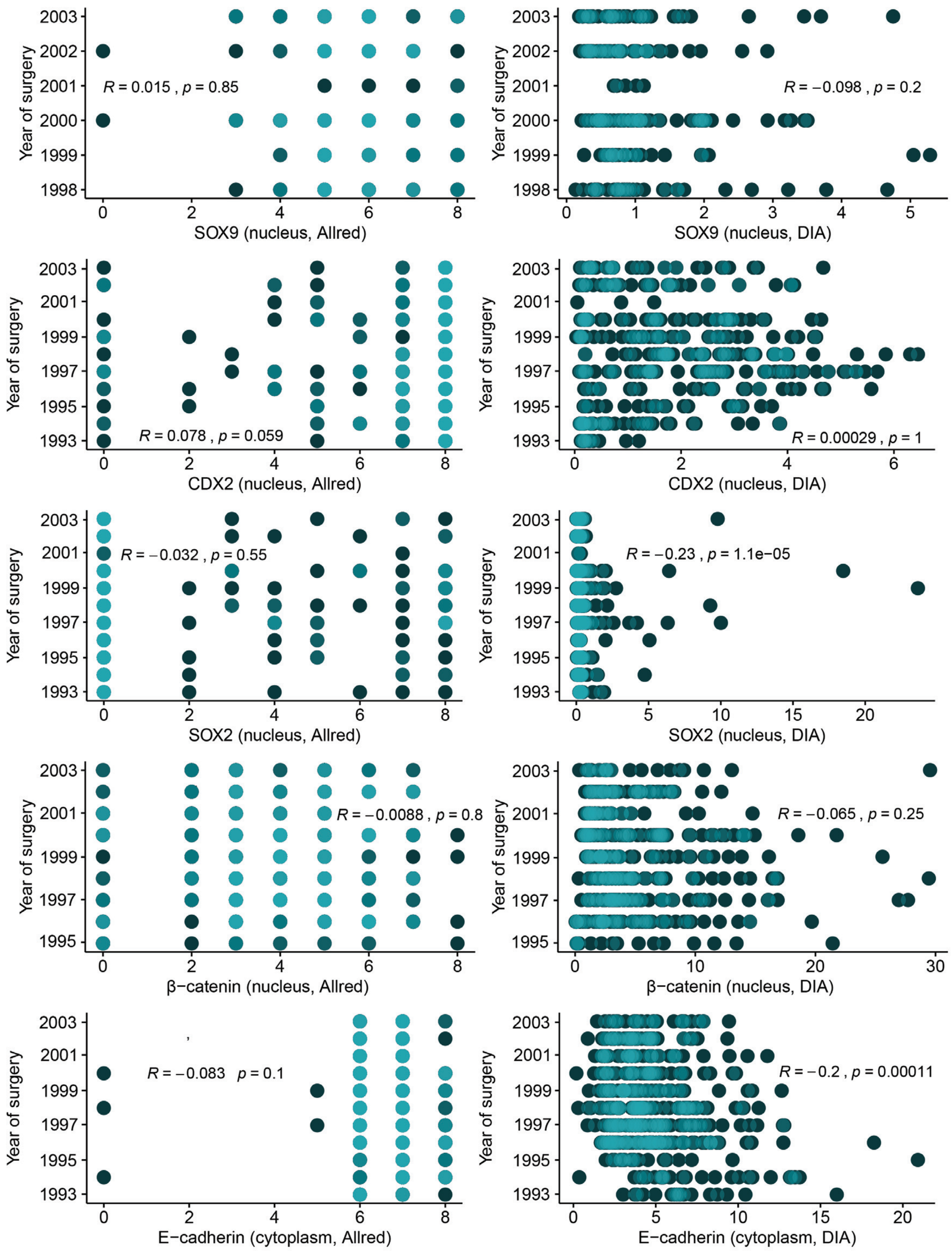


**Figure S3** | Relationship between staining and sample age. R-values represent Spearman correlation coefficients. Abbreviations: DIA, digital image analysis.

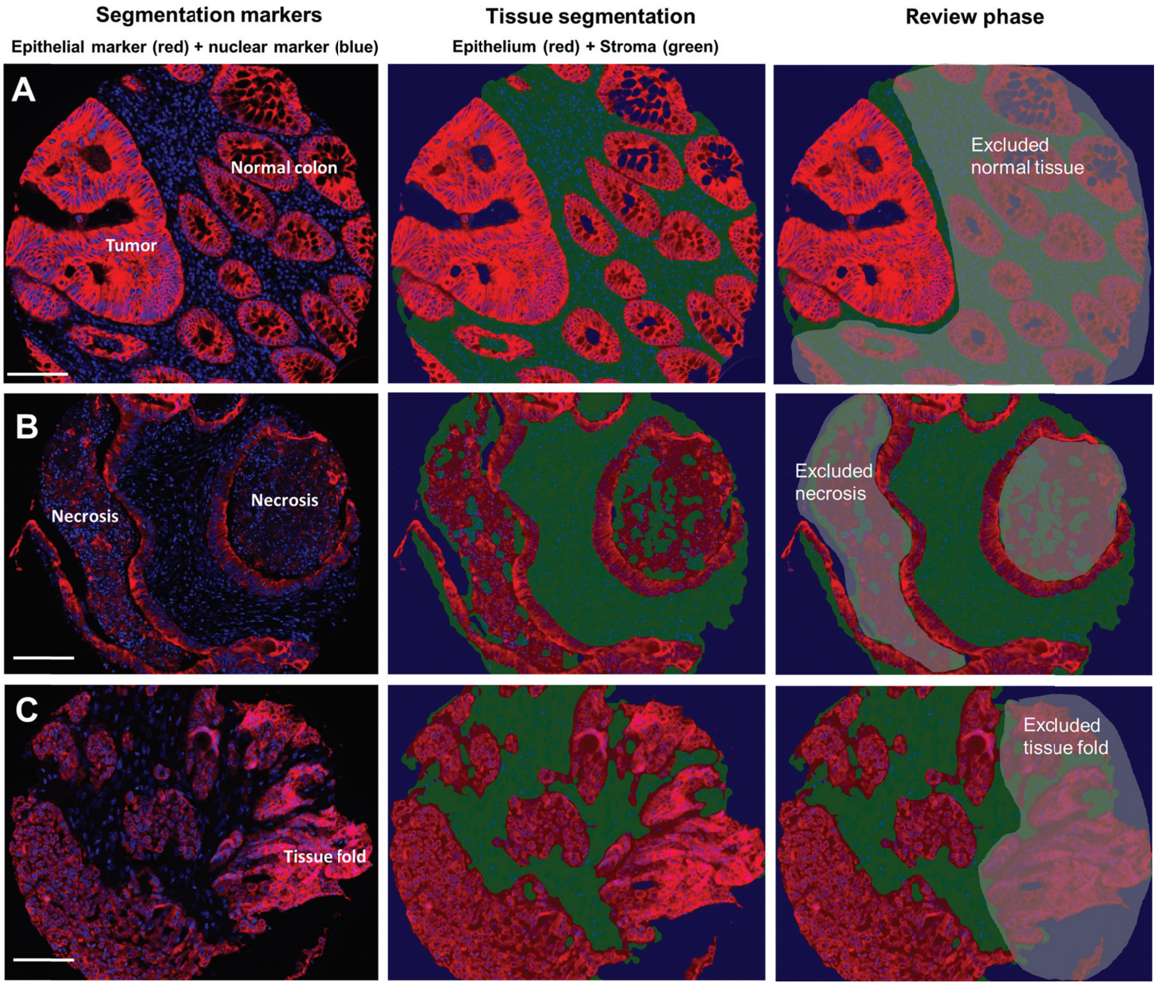


**Figure S4 |** Illustration of manual curation of tissue cores with normal colon glands (A), necrotic areas (B) and tissue folds (C). Scale bar, 0.1 mm.

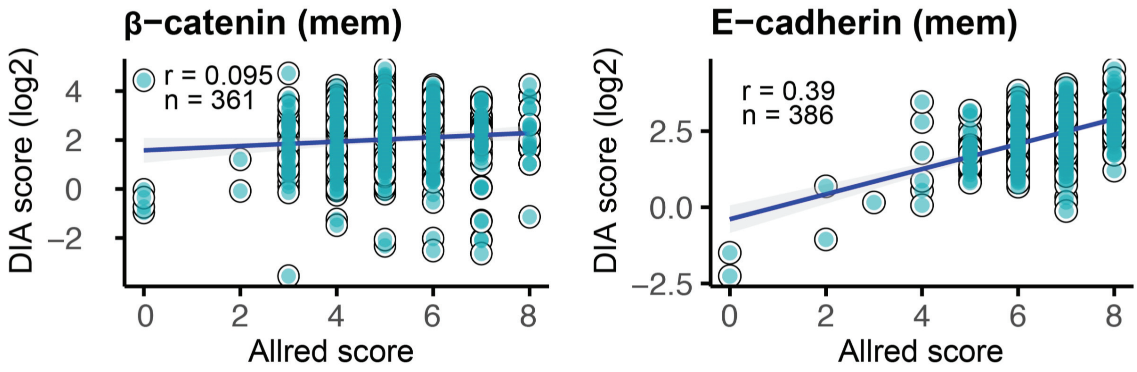


**Figure S5** | Correlation between Allred and DIA scores for β-catenin and E-cadherin membrane staining. Correlation coefficients were calculated using the Spearman's rho method. DIA scores were log2 transformed for visualization. Abbreviations: mem, membrane; DIA, digital image analysis.

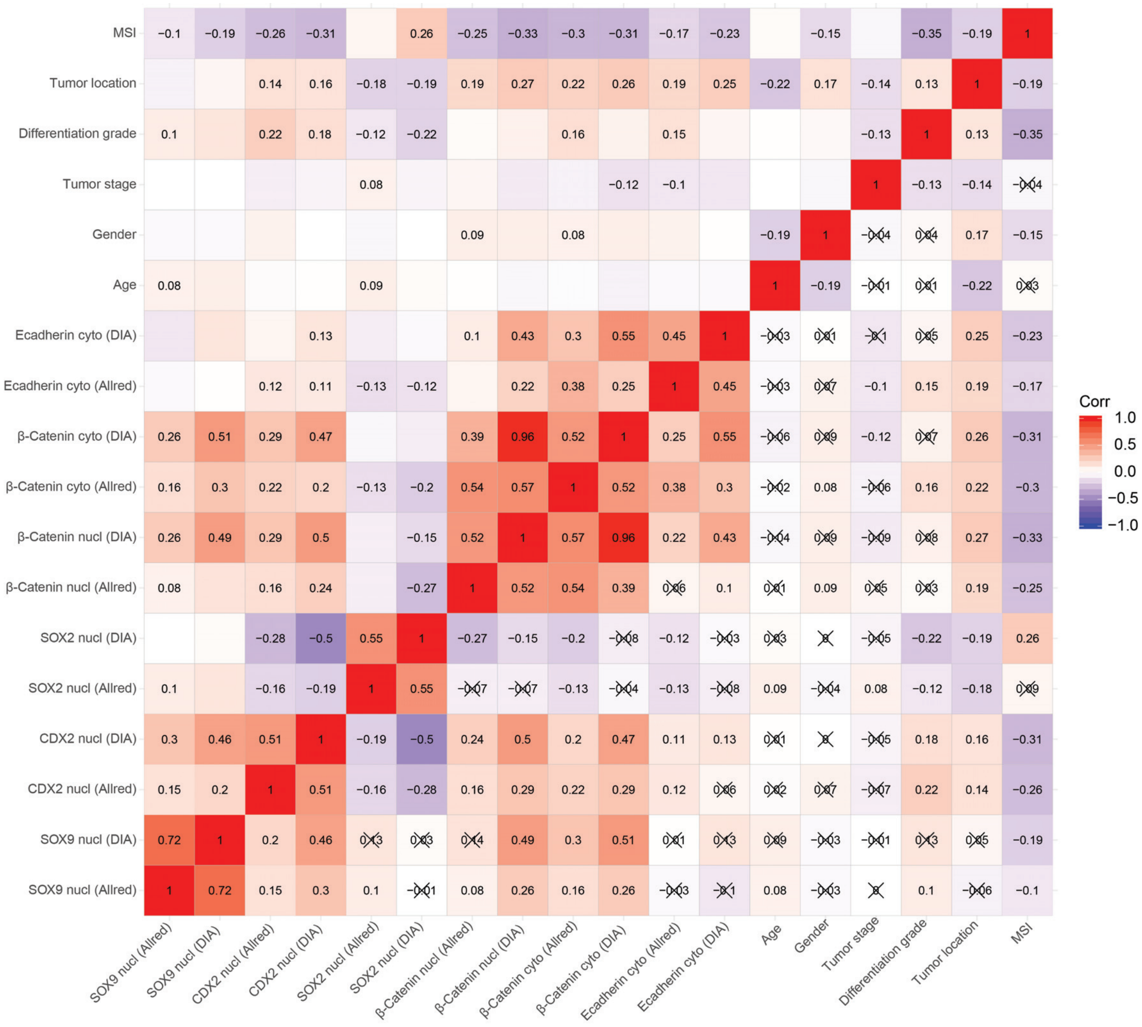


**Figure S6** | Correlation matrix for stained markers and clinical variables. Values represent Spearman correlation coefficients. Tumor locations were grouped into right, left, rectum and synchronous. Differentiation grade was grouped into high, moderate, low and mucinous. Abbreviations: DIA, digital image analysis, Corr, correlation, MSI, microsatellite instability; nucl, nuclear; cyto, cytoplasm. X indicates non-significant values with correlation p-value > 0.05. Non-significant values are omitted from the upper matrix to emphasise main results.

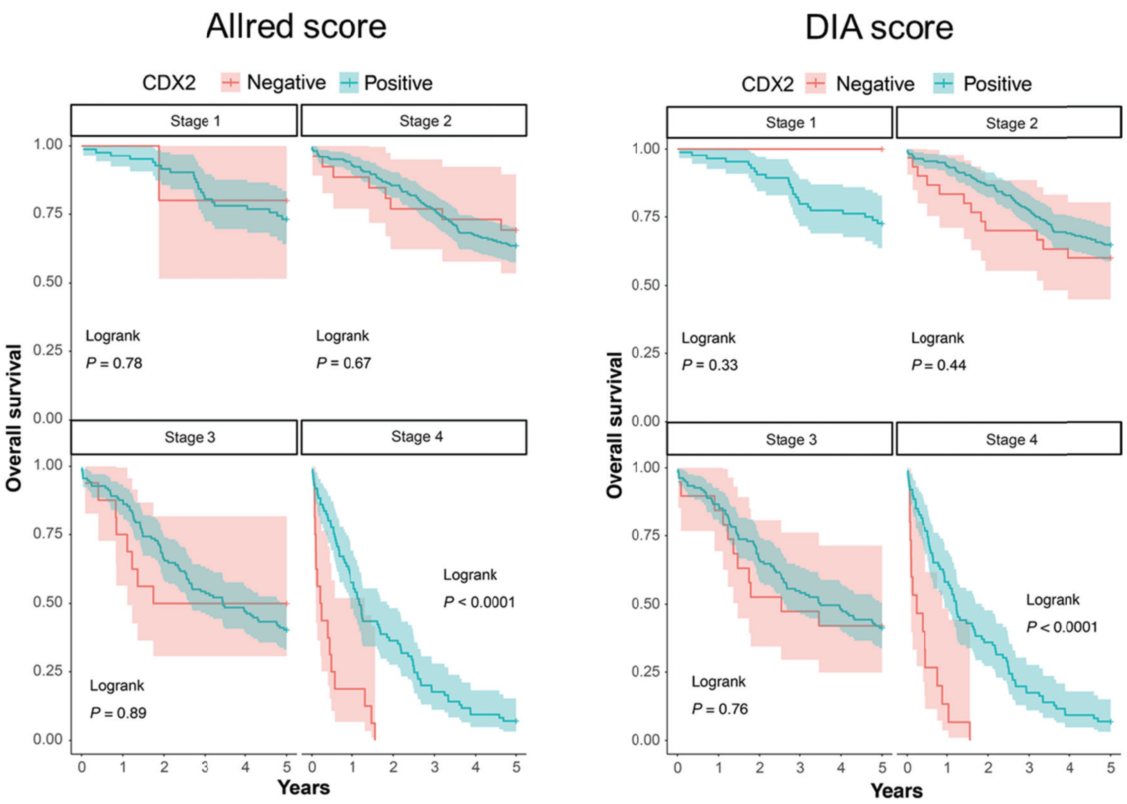


**Figure S7** | Both DIA and Allred scoring are able to detect the strong prognostic value of CDX2 in colorectal cancer stage IV. Thresholding was performed using all cases with information on CDX2. Survival analyses include only cases with both Allred score and DIA score data (n = 589). Abbreviation: DIA, digital image analysis.

| Biomarkers | Cycle 1 | Cycle 2 | Cycle 3 | Cycle 4 | Cycle 5 |
|---|---|---|---|---|---|
| **SOX9** | SOX9 (Opal 570) | Epi-cocktail (Opal 670) | DAPI | - | - |
| **E-cadherin** | E-cadherin (Opal 570) | Epi-cocktail (Opal 670) | DAPI | - | . |
| **β-catenin** | β-catenin (Opal 570) | Epi-cocktail (Opal 670) | DAPI | - | - |
| **CDX2/SOX2** | CDX2 (Opal 520) | SOX2 (Opal 570) | Epi-cocktail (Opal 670) | DAPI | - |
| **CDX2/Marker 2/ Marker 3** | CDX2 (Opal 520) | Marker 2 (Opal 570) | Marker 3 (Opal 620) | Epi-cocktail (Opal 690) | DAPI |

**Table S1** | Overview of Opal staining procedure for each biomarker analysed in the study. Marker 2 and 3 indicate unpublished biomarkers not included in the study. Abbreviation: Epi, Epithelial.
